# Acute Ischaemic Stroke: Therapeutic Targets and Key Modulators of the Fibrinolytic System and Clot Formation

**DOI:** 10.1007/s12975-026-01502-0

**Published:** 2026-10-13

**Authors:** Oshi Swarup, Anna H. Balabanski, Geoffrey C. Cloud, Robert L. Medcalf

**Affiliations:** 1 Department of Neurology, Alfred Hospital, Melbourne, 3004 Australia; 2 Department of Clinical Neurosciences, School of Translational Medicine, Monash University, Melbourne, Victoria Australia; 3 Department of Neurology, Melbourne Brain Centre at the Royal Melbourne Hospital, University of Melbourne, Parkville, Australia; 4 Australian Centre for Blood Diseases, School of Translational Medicine, Monash University, Melbourne, Victoria Australia

**Keywords:** Ischemic stroke, Fibrinolysis, Thrombolysis, Plasminogen activation, Adjunctive treatment

## Abstract

The processes of clot formation and clot dissolution with fibrinolysis underpin the pathophysiological mechanisms of acute ischaemic stroke (AIS) and its treatment. Detailed understanding of the modulation of this system is required to develop effective and safe stroke therapeutics. Clot formation occurs with the interaction of the coagulation cascade, along with platelets and fibrin meshwork. Conversely, fibrinolysis involves the serine protease plasmin, its zymogen, plasminogen and its enzymatic conversion by tissue plasminogen activator. The regulators and inhibitors that modulate the interactions in clot formation and fibrinolysis can be potential targets for new and emerging stroke therapies. The aim of this narrative review is to help inform stroke clinicians and researchers about the role of the fibrinolytic system and its inhibitors in AIS and the potential for new therapeutic targets. We outline the processes involved in clot formation and dissolution, the role of endogenous inhibitors of the fibrinolytic system, followed by potential therapeutic targets including tissue plasminogen activators as well as novel therapies and adjuncts. Better understanding of these processes will provide insight into the variable response to current stroke fibrinolytics amongst AIS patients and help to develop new and improved AIS therapeutics in future.

## Introduction

Ischaemic stroke is caused by obstruction to the blood supply to a particular part of the brain resulting tissue infarction due to localised thrombus formation or thromboembolism from a distant site. This can be due to a myriad of different mechanisms. Thrombus formation may occur locally on an underlying atherosclerotic plaque or other vascular abnormalities such as arterial dissection or vasculitis. However, it can also occur due to formation of a thromboembolism distant to the site of stroke. The most common of which is cardio-embolism or clots originating from the heart, for instance related to atrial fibrillation [1]. The processes of clot formation and dissolution are two sides of the same coin and ultimately underpin the pathophysiological mechanisms of acute ischaemic stroke and its treatment. In this review we set out the current understanding of the underlying mechanisms of thrombosis and fibrinolysis applicable to AIS. The aim of this narrative review is to help inform stroke clinicians and researchers about the role of the fibrinolytic system and its inhibitors in AIS and the potential for new therapeutic targets. Here we explore the processes involved in clot formation and dissolution, the role of endogenous inhibitors of the fibrinolytic system, followed by potential therapeutic targets as well as novel therapies and adjuncts. To address the question as to how we can identify new potential therapeutics and targets from our understanding of the fibrinolytic system in acute ischaemic stroke, a literature review was undertaken using Ovid MEDLINE database, article abstracts to identify relevant articles, with full text review of included articles. Further articles were selected from reference list of selected articles. Given the nature of this review, targeted publications were selected based on their relevance to the topic rather than a systematic review of the literature.

## Pathophysiology of Clot Formation and Fibrinolysis in AIS

The pathogenesis of acute ischaemic stroke is commonly occlusive clot formation in vivo. Thrombus formation begins with platelet activation and aggregation, followed by initiation of the coagulation cascade which eventuates in fibrinogen being converted to fibrin by thrombin, leading to the formation of a fibrin mesh. Red blood cells and platelets initially trapped in this mesh become more compact as further fibrin deposition occurs. Platelet crosslinking with fibrin and the assistance of von Willebrand Factor (vWF), pulls the fibrin meshwork and leads to clot retraction, compressing the involved red blood cells [2–4]. Activated factor XIII (FXIIIa) further stabilises the clot via the crosslinking of fibrin molecules [5]. Activated platelets propagate the inflammatory reaction, leading to infiltration of inflammatory cells into the mesh. These cells include neutrophils, which release neutrophil extracellular traps (NETs) that encase the thrombus mesh [6].

**Fig. 1:**
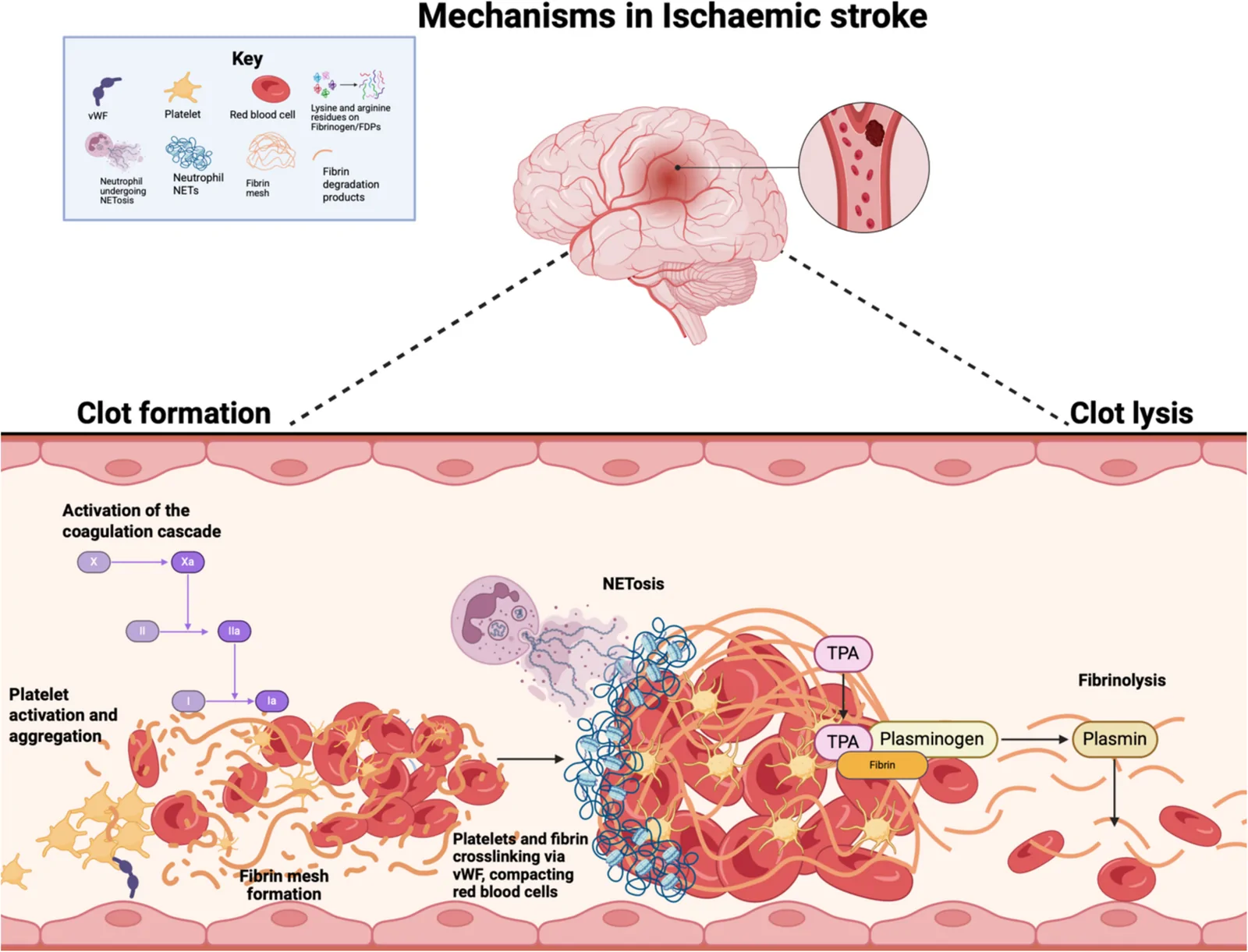
Summary of the mechanisms of clot formation and clot dissolution in acute ischaemic stroke (Created in BioRender. Swarup, O. (2026) https://BioRender.com/tyx25i)

**Fig. 2:**
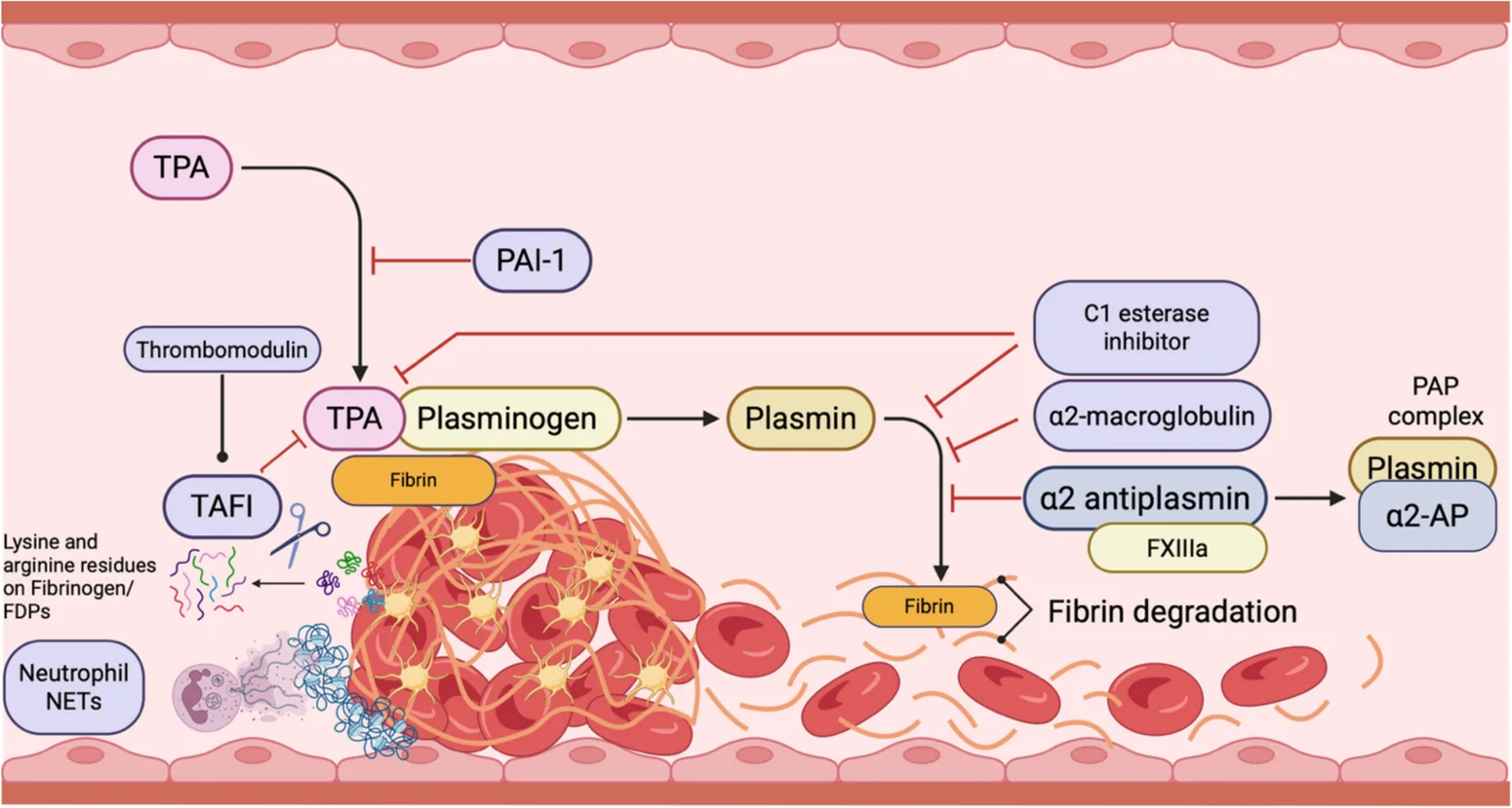
Summary of endogenous inhibitors of the fibrinolytic system. (Created in BioRender. Swarup, O. (2026) https://BioRender.com/s21l460)

Conversely, fibrinolysis underpins the process of clot dissolution and is the mainstay of current pharmacological reperfusion therapies for acute ischaemic stroke. The key factors involved in clot dissolution or fibrinolysis in acute ischaemic stroke are the serine protease plasmin, its zymogen, plasminogen and its enzymatic conversion by tissue plasminogen activator (tPA). Upon thrombus formation, tPA in endothelial cells is released into the bloodstream. tPA binds to fibrin and plasminogen, forming a plasminogen-tPA-fibrin complex, to activate plasminogen to plasmin. It is the enzymatic cleavage of plasminogen by tPA into plasmin, which in turn cleaves the crosslinked fibrin in the clot into fibrin degradation products (FDPs). It is this activation of plasminogen to plasmin by tissue plasminogen activators (tPA) which forms the mainstay of stroke treatment at present (Figs. 1 and 2). Understanding clot formation and dissolution by fibrinolysis, allows us to better understand mechanisms behind acute ischaemic stroke and helps to identify potential treatment targets.

## Endogenous Inhibitors of Fibrinolysis

While it is the activation of the fibrinolytic system that underpins current acute ischaemic stroke treatment, there are several breaks and checks in this system which modulate patient responses. It is important to understand the roles of these key modulators as they can have a significant effect on patient response to stroke therapeutics. There are several inhibitors in the blood and within the blood clot itself that regulate the fibrinolytic system by their effect on tPA activity (Fig. 2; Table 1). The best characterised inhibitor is plasminogen activator inhibitor-1 (PAI-1), which complexes with tPA and is rapidly eliminated via the liver over 5 min [7]. PAI-1 is produced in the liver, adipose tissue, endothelial cells, platelets and megakaryocytes, but the plasma level of PAI-1 is relatively low [7]. On the other hand, PAI-1 is present in large amounts in platelets and so platelet derived PAI-1 becomes an important impediment to thrombolysis. While platelet PAI-1 certainly slows the process down, the latency of PAI-1 needs to be considered as it is not always in an active state and becomes inactive within 2 h owing to its short plasma half-life [8, 9]. In contrast, alpha-2‑antiplasmin (α_2_AP) is the most abundant inhibitor of plasmin in plasma, circulating in significantly higher concentrations compared to other inhibitors [10]. α_2_AP crosslinks to fibrinogen and fibrin via activated factor XIII (FXIIIa), tethering it to the clot and making it resistant to fibrinolysis. Further, in plasma, lysine residues in the C-terminal region of α_2_AP bind to lysine binding sites on plasmin leading to a plasmin-mediated conformational change producing a strong covalently bound plasmin-alpha2-antiplasmin (PAP) complexes [10]. Thrombin activatable fibrinolysis inhibitor (TAFI) or its activated carboxypeptidase form TAFIa, is another regulator of fibrinolysis. It does this by cleaving the lysine and arginine residues on fibrin and fibrin degradation products (FDPs) at the carboxy terminal which are exposed during plasmin mediated breakdown. By removing these residues, TAFIa weakens the ability of plasminogen to bind to fibrin and to initiate plasminogen activation, thereby sparing clots from premature fibrinolysis and leaving plasmin to form PAP complexes. The clinical relevance of TAFI has been explored in the prospective Ludwigshafen Risk and Cardiovascular Health (LURIC) study [11]. This study found that the TAFI Thr325Ile allele was associated with increased incidence of stroke and lower age of onset, suggesting TAFI has a key pathophysiological role in stroke [11]. This is further corroborated by the finding that elevated functional TAFI levels have been associated with an increased stroke risk [12].

Thrombomodulin is another modulator of fibrinolysis and extends lysis time. It exists as a transmembrane protein in vascular endothelial cells, which is cleaved by neutrophil proteases and metalloproteases, and left in a fragmented form in plasma which in turn slows down fibrinolysis. It does this by binding to thrombin forming Thrombin-Thrombomodulin complexes which leads to TAFI activation, thereby reducing fibrinolysis. Other inhibitors of plasmin and as such fibrinolysis, include alpha2-macroglobulin, C1-esterase inhibitor, and alpha-1- antitrypsin, however, these are less well characterised as potential targets in stroke [13](Fig. 2; Table 1). These notable fibrinolytic system modulators that influence the individual patient response to tissue plasminogen activators used in hyperacute stroke treatment may be useful markers in future development of stroke therapeutics.

## Clot Composition

Alongside understanding potential targets in the fibrinolytic system, it is important to understand the basis of clot composition. Clot composition has been studied extensively ex-vivo from patients with acute ischaemic stroke via collection through endovascular thrombectomy. Immunohistochemistry of thrombectomy clots has shed light on many aspects of clot composition, origins, response to treatment and imaging correlations in acute ischaemic stroke. A meta-analysis of 22 studies which analysed histopathology of thrombectomy clots and associated outcomes, revealed that red blood cell rich clots were associated with hyperdense vessel sign, had shorter procedure times, and better angiographic outcomes than fibrin or platelet rich clots [14]. Further, red blood cell rich clots have been associated with a greater response to thrombolytic therapy, thought likely to be related to looser clot architecture, with enlarged pores in the ultrastructure of the clot in other studies [15]. Although this meta-analysis did not reveal correlation of clot composition to stroke aetiology, there have been several other studies assessing this aspect. This has been done through the assessment of the quantitative fractions of red cell, fibrin/platelet and white cell components of retrieved thrombectomy clots to assess the association with different stroke characteristics and aetiologies [16, 17]. These assessments support the finding that clots from a cardioembolic source had a significantly higher fibrin and platelet as well as white cell content compared to non-cardioembolic clots which had a higher red cell fraction. Further, a cut off 60% Fibrin/Platelet components had a positive predictive value of 80.7% in identifying cardioembolic strokes in this study, suggesting a potential for determining thrombus aetiology based on fractional components and as such impacting secondary prevention choices. Interestingly however, DNA content within clots has also been used to discriminate between cardioembolic and atherosclerotic clots, finding cardioembolic clots having a higher DNA content on biochemical analysis of homogenates of retrieved thrombectomy clots [18]. It is because of this heterogeneity in clot composition relating to different aetiologies of AIS, that we can understand why there is variability in responses to treatment in stroke. While there is substantial heterogeneity in the mechanism and tempo of thrombus formation, the shared pathways underpinning thrombosis and fibrinolysis are integral to our understanding of AIS and its therapeutic targets.

**Table 1:** Summary of the inhibitors of fibrinolysis

Inhibitor	Mechanism
PAI‑1 (plasminogen activator inhibitor‑1)	Complexes with tPA, leading to rapid clearance.
α2AP (Αlpha-2‑antiplasmin)	Covalently crosslinks to fibrin via FXIIIa and binds plasmin, forming plasmin‑α2AP complexes
TAFI/TAFIa (thrombin‑activatable fibrinolysis inhibitor)	Removes C‑terminal lysine/arginine residues on fibrin, reducing the ability of fibrin to bind to plasminogen.
Thrombomodulin	Forms thrombin‑thrombomodulin complex to activate TAFI, reducing fibrinolysis. Activates the protein C pathway, reduces thrombin generation, may indirectly promote fibrinolysis
Alpha‑2‑macroglobulin	Inhibits plasmin, and kallikrein, limiting fibrinolysis.
C1‑esterase inhibitor	Inhibits plasmin, and kallikrein, limiting fibrinolysis.
Alpha‑1 antitrypsin	Inhibits plasmin and other serine proteases

## Therapeutic Targets

**Fig. 3:**
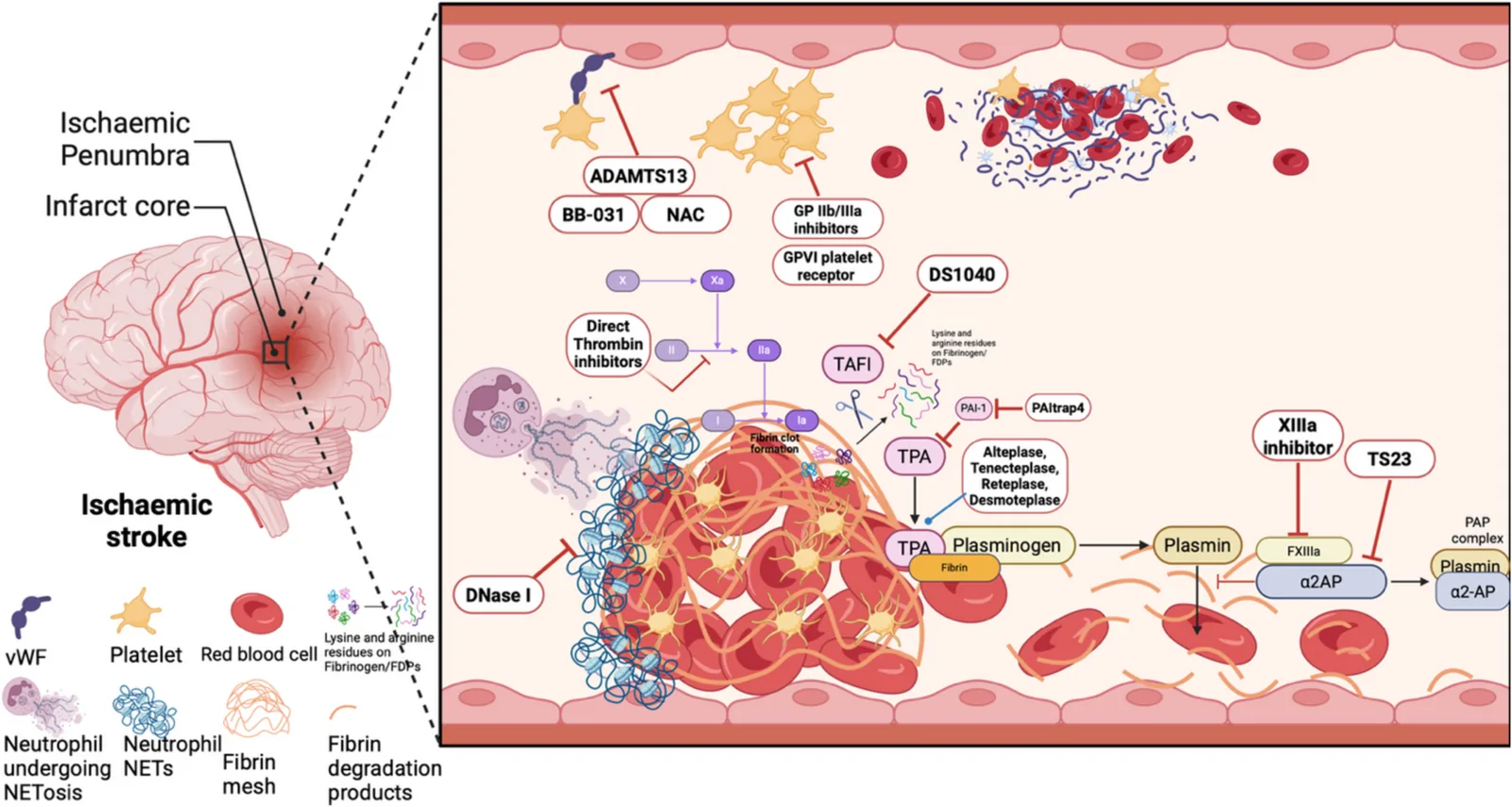
Current and future fibrinolytics and adjuncts with their mechanisms of action in acute ischaemic stroke. (Created in BioRender. Swarup, O. (2026) https://BioRender.com/whqyczl)

Conversion of the plasma zymogen plasminogen into its fibrinolytic form, plasmin, by plasminogen activating proteases is the principle behind therapeutic thrombolysis. While there has been considerable evolution in reperfusion strategies for AIS with the advent of endovascular thrombectomy, thrombolysis remains integral to hyperacute stroke management both as bridging therapy in combination with thrombectomy for large vessel occlusion strokes and as primary treatment for more distal or small intracranial vessel occlusions for eligible patients as per current guidelines [19]. The actions of fibrinolytics and adjuncts in the fibrinolytic and coagulation pathways are discussed in this section (Tables 2 and 3; Fig. 3). 

## Development of Plasminogen Activators for Thrombolysis

The first generation of lytics were Streptokinase and Urokinase, both of which had lower fibrin specificity compared to later lytic agents. Subsequently, recombinant tissue-type plasminogen activator (tPA), a more fibrin targeted protease, has driven acute stroke treatment for nearly three decades. Recombinant tPA, Alteplase, was developed in 1982, with its initial utility being in acute myocardial infarction, and became approved for clinical use for acute ischaemic stroke in 1996 by the U.S. Food and Drug Administration (FDA) following the landmark National Institute of Neurological Disorders and Stroke rt-PA Stroke Study (NINDS) study [20, 21]. Since then, several tissue plasminogen activators have been explored including Tenecteplase, Reteplase, Desmoteplase, Pamiteplase, Monteplase, Lanoteplase and Pro-urokinase (Table 2).

Alteplase is noted to have reduced half-life of approximately 5 min and is administered as an intravenous bolus followed by infusion over 1 h at 0.9 mg/kg dosing [22]. Tenecteplase [23] has an extended half-life (approximately 22 min), improved fibrin selectivity compared to fibrinogen and resistance to inhibition by PAI-1 [24] although the degree of PAI-1 resistance of TNK was recently reported to be reduced in its fully active two-chain form [25]. The extended plasma half-life of TNK permitted its delivery as a single bolus dose compared to Alteplase [26]. Another recombinant tPA derivative being explored is Reteplase (rPA), a truncated variant of tPA with an extended half-life (approximately 18 min), ease of administration as a double bolus of 10 units each, but with lower fibrin specificity [27]. Tenecteplase is the main contender in stroke practice with several recent trials and now a meta-analysis suggesting efficacy and ease of use compared to Alteplase while Reteplase is also now emerging with clinical trial evidence demonstrating efficacy and safety [28, 29]. More recently, Reteplase has also been compared to standard of care Alteplase within the 4.5-hour window for thrombolysis in stroke. In an open label randomised clinical trial [30], Reteplase was more likely to result in an excellent functional outcome (modified Rankin scale 0–1) compared to Alteplase, with a risk ratio of 1.13 ((*P* < 0.001 for noninferiority and *P* = 0.002 for superiority) [30]. While the prespecified primary safety outcome of symptomatic ICH in the first 36 h was similar, 2.4% of patients in the Reteplase arm compared to 2% in the Alteplase arm (risk ratio (RR) 1.21; 95% CI 0.54–2.75), notably there were higher rates of adverse events (RR 1.11; 95% CI 1.03–1.20) and higher incidence of any ICH at 90 days (RR 1.59; 95% CI 1.00-2.51) with Reteplase compared to Alteplase [30]. As such, further blinded trials are needed to assess the outcomes in comparison to Alteplase and Tenecteplase [31]. Other relevant thrombolytics studied include the original bat derived protease, Desmoteplase which had been trialled in the DIAS 3 and 4 trials in the extended thrombolysis time window in up to 9-hour window against placebo [32, 33]. It was shown to be safe but did not demonstrate a significant functional improvement. However, the results of these studies were limited by trial design in terms of imaging and patient selection. It was completed prior to the extensive understanding of CT perfusion imaging-based patient selection for AIS in the extended time window up to 24 h [34–37]. More recently, intravenous recombinant human Pro-urokinase (rhPro-UK) has re-surfaced as potential stroke therapy since its initial use as an intra-arterial agent being superseded by endovascular thrombectomy [38]. It has some potential advantages over current thrombolytics, due to its theoretically reduced systemic activation from its proenzyme form to Urokinase, lending to reduced risk of haemorrhage as well as being a lower cost thrombolytic compared to Alteplase and Tenecteplase [39, 40]. rhPro-UK has been evaluated recently in the Phase 3 open label PROST-2 Trial in patients ineligible or refusing thrombectomy, demonstrating non-inferiority to Alteplase in safety and functional outcomes, with a lower rate of intracranial haemorrhage [39]. Further metanalysis of randomised controlled clinical trials supports its non-inferiority to Alteplase in terms of functional outcomes, and safety, however larger trials are still needed [41]. Similarly, fibrinolytic agents with higher fibrin selectivity and hence, lower systemic plasminogen activation have additional advantages with a higher theoretical safety profile and targeted action. One such agent, which has also similarly resurfaced more recently in Russia, includes a low immunogenic recombinant Staphylokinase given as a rapid single bolus dose of 10 mg. In the FRIDA trial, low immunogenic recombinant Staphylokinase demonstrated non-inferiority to Alteplase in the 4.5-hour time window for AIS, with findings further corroborated by the FORPI registry observational data [42, 43]. Other agents, that have been studied include JX10, a small molecule derived from the fungus S. microspora, which facilitates fibrinolysis by inducing a conformational change in plasminogen from closed to open. This was studied in a phase 2a randomised double blinded placebo-controlled trial, in patients 12 h from last known well in patients not able to have tPA or thrombectomy [44]. It was well tolerated, and had a finding of improved functional outcomes in patient’s ineligible for current guideline-based therapies, however further larger phase 3 studies need to be conducted to derive further conclusions about efficacy.

**Table 2:** Summary of the key differences between different thrombolytic agents

Agent	Fibrin specificity	PAI-1 resistance	Dosing/Administration	Clinical notes
In clinical use				
Alteplase	Moderate	Low	IV bolus + 1 h infusion (0.9 mg/kg)	Standard of care, well‑established
Tenecteplase	High	High	Single bolus (0.25 mg/kg)	Standard of care, Similar efficacy to Alteplase; easier administration;
Emerging/previously studied				
Reteplase	Lower than Alteplase/TNK	Moderate	Two boluses (10 units each)	Emerging data in stroke (RAISE trial)
Pro-Urokinase	High	Moderate	IV 15 mg bolus + 20 mg infusion over 30 min	Emerging trials (PROST-2); non-inferiority to Alteplase, signal to lower ICH risk
Desmoteplase	High	Very high	Single IV bolus 90 µg/kg	Previous clinical trials DIAS 3 and 4; Safe but did not improve functional outcome.
Low Immunogenic Recombinant Staphylokinase	High	Unknown	10 mg IV bolus	Emerging trials: FRIDA Trial; non-inferiority to Alteplase. Supporting Clinical registry data

Whilst there are several thrombolytics that are under development and have been studied, the mainstay of stroke thrombolysis remains with Alteplase or Tenecteplase for most centres [19]. Current stroke treatments involve recanalization by means of thrombolysis with or without thrombectomy. While thrombolysis is only effective in clot dissolution or recanalization in approximately 30% of cases within a licenced therapeutic window under 4.5 h or in the extended time window for selected cases, thrombectomy relies on specialist quaternary centres and favourable imaging findings [45]. However, the proportional treatment benefit in terms of disability free survival decreases further away from symptom onset [46]. The role for thrombolysis remains relevant and important even with the advent of thrombectomy, as the time window of treatment has been extended further for select cases with evidence emerging for treatment up to 24 h which has been included in the American Heart Association/American Stroke Association 2026 guidelines [19]. Further, thrombolysis remains especially important in the setting of medium and small intracranial vessel occlusions and in the instances where endovascular thrombectomy may fail. As such identifying a suitable therapeutic target for thrombolysis remains the mainstay of treatment in acute ischaemic stroke. 

## Adjuvant and Novel Therapies

While the current mainstay of stroke management is with tissue plasminogen activators (tPA), there are several emerging targets in varying stages of research as adjuncts or primary treatment targets within the fibrinolytic system (Table 3; Fig. 3).

### Adjunctive Antiplatelet/Anticoagulants

To date, there have been several clinical trials assessing the adjunctive addition of antiplatelet and anticoagulants to thrombolysis [47]. Intravenous heparin and intravenous aspirin were studied in the open-label, multicentre, randomized controlled trial, MR CLEAN-MED as an adjunct to endovascular thrombectomy. This study found no notable clinical benefit, at the expense of increased risk of haemorrhage in those receiving IV aspirin or heparin as adjunctive treatment [48]. Similarly, the ARTSS trial testing the direct thrombin inhibitor, Argatroban with tPA for acute ischemic stroke, while finding greater efficacy in recanalization, found higher incidence of ICH [49, 50].

Similarly, platelet inhibition has also been explored as an adjunct to stroke treatment. The ARTIS trial evaluating intravenous aspirin as an adjunct to thrombolysis was also prematurely terminated due to a higher rate of sICH [51]. Other platelet targets have emerged since including GPVI platelet receptor which is a collagen adhesion receptor. This has been identified to have increased expression in patients with acute ischaemic stroke, making it a suitable biomarker and target. GPVI monoclonal antibody called Glenzocimab has also been investigated in combination with IVT in ACuTe Ischemic Stroke Study Evaluating Glenzocimab Used as Add-on Therapy Versus placEbo (ACTISAVE) trial, finding no difference in the primary outcome which was functional outcome at 90 days on follow up between arms [52, 53]. Similarly, preliminary results of the GREEN trial, evaluating the efficacy and safety of Glenzocimab as an adjunct to thrombectomy was stopped early due to futility as well [54].

While adjunctive platelet inhibition and anticoagulation have not yielded improved clinical outcomes and come at a cost of higher haemorrhagic risks, the GP IIb/IIIa receptor blockers such as Tirofiban have shown some promise in adjunctive platelet inhibition. There have been a few trials that have evaluated the adjunctive use of tirofiban compared to placebo as an adjunct to thrombectomy and thrombolysis [55–57]. Although the initial RESCUE- BT trial initially found no clinical or recanalization benefit, with the addition of higher rates of asymptomatic ICH, there was a signal for improved functional outcomes in patients with large artery atherosclerosis [55, 57, 58]. Other studies have assessed tirofiban adjunctive to thrombectomy for large vessel occlusion, finding that given pre-thrombectomy improved the proportion of patients with first pass recanalization [59]. More recently, the preliminary results from the ATTRACTION trial, assessing the use of adjunctive tirofiban post thrombectomy further supported improved functional outcomes, without significant increase in ICH. Subsequent phase 3 randomised controlled clinical trial ASSET-IT found adjunctive use of Tirofiban after thrombolysis without thrombectomy demonstrated an improvement in likelihood of an excellent functional outcome (modified Rankin Scale (mRS) score of 0–1 at 90 days) in patients with non-cardioembolic stroke albeit with a higher rate of sICH compared to placebo [60]. This is further corroborated by the INSTANT trial, favouring adjunctive tirofiban with TNK in non-large vessel occlusion (LVO) patients with inadequate lytic response [61]. While recent trials with tirofiban have shown promise, another agent targeting the same pathway, Eptifibatide, initially demonstrated some promise in earlier trials however did not have an improved functional outcome as a lysis adjunct in the more recent phase 3 clinical trial, the MOST study [62]. This compared eptifibatide, Argatroban vs. placebo as lysis adjuncts to thrombolysis with or without thrombectomy, finding no difference in functional outcomes, and increase risk of mortality in the Argatroban arm.

### Targeting Neutrophil Extracellular Traps (NETs)

More recently, neutrophil extracellular traps (NETs) have been postulated as a major factor impacting tPA resistance in stroke treatment. During clot formation, neutrophils are attracted to the area of thrombosis, whereby they release Neutrophil Extracellular Traps (NETs). NETs are web-like structures composed of chromatin, granular antimicrobial proteins and citrullinated histone H3 released by neutrophils during a process called NETosis, which is a unique form of cell death [6, 63]. While this was originally identified as a mechanism to trap and kill pathogens, these nets have been identified on clot surfaces, which in turn create a net for trapping platelets and red blood cells leading to lysis resistance [64]. In histopathological studies, NETs were seen predominately in the outer shell of the clot, creating a barrier for tPA in accessing clot bound plasminogen conferring additional tPA resistance [65].

Dornase alfa, a recombinant human deoxyribonuclease I, can potentiate tPA-mediated clot lysis via breakdown of neutrophil extracellular traps and can be considered as a potential therapeutic target in stroke [6]. Previous studies have demonstrated that the addition of DNase 1 ex-vivo, to clots obtained from endovascular thrombectomy procedures in patients with acute ischaemic stroke, potentiated clot lysis in combination with tPA compared to tPA alone as measured by clot weight reduction [6, 65]. This has been further evaluated in mouse studies, demonstrating DNase-1 in combination with tPA compared to tPA alone improved outcomes [66]. As such, NETs are emerging as an important treatment target in acute ischaemic stroke treatment, with Dornase alfa being evaluated as an adjunctive treatment to thrombolytics. This has recently been evaluated in, EXTEND-IA DNase (NCT05203224) a phase 2 clinical trial recently completed with preliminary results demonstrating safety (final publication awaited at time of writing) in multiple centres across Australia evaluating DNAse-1 with Tenecteplase, which may inform phase 3 trials [67].

### Von Willebrand Factor

Von Willebrand factor (vWF) is a glycoprotein that has several roles in stroke pathogenesis. Upon vascular injury, vWF binds to exposed collagen type I and II on vessel walls, leading to a conformational change that exposes platelet glycoprotein (GP)Iba binding sites thereby leading to platelet adhesion and activation at the outset of clot formation. It has been studied previously in its role in acute myocardial infarction and more recently in the pathogenesis of acute ischaemic stroke. In the Rotterdam Study, 197 ischaemic strokes occurred among 6,250 participants over five years, with higher von Willebrand factor levels associated with increased stroke risk [68]. On analysis of clots retrieved by endovascular thrombectomy, vWF was found on average composing of 20.3% of the clot, making this a very viable target in stroke research [69].

ADAMTS13, a protease involved in vWF cleavage, has been evaluated as a possible adjunctive treatment in acute ischaemic stroke. A study assessing patients with AIS undergoing thrombectomy for vWF and ADAMTS13 levels demonstrated higher vWF level and vWF: ADAMTS13 ratios in patients with severe stroke syndromes (National Institutes of Health Stroke Scale (NIHSS) score > 15) and found that elevated plasma vWF correlated with thrombus vWF, platelet and fibrin levels [70]. As such, ADAMTS13 as a therapeutic target was evaluated in mouse models with middle cerebral artery (MCA) stroke from vWF-containing thrombi in a study comparing effect of ADAMTS13 administration to tPA [69]. They found that ADAMTS13 compared with tPA was more effective at recanalization and reduced infarct sizes in mouse models with no instances of haemorrhagic transformation, leading to this being another potentially useful treatment target in stroke. However, in this study ADAMTS13 was compared directly to tPA, rather than as an adjunct in combination with tPA. This posed a limitation in this study as the clots studied were vWF rich and red blood cell poor, making the result less generalisable to in vivo clots of varying compositions. Similar results have been found in other animal studies of ADAMTS13 compared to tPA, which demonstrated no haemorrhages compared tPA treatment and so a more favourable safety profile [71]. In further mouse models, a constitutively active ADAMTS13 has been explored as a therapeutic option in acute ischaemic stroke, as the native protein requires a conformational change to be active. Overall finding was that this constitutively active ADAMTS-13 had higher recanalization rates than he native moleculein these models [72]. In fact, ADAMTS13 appears to have a protective effect on the blood brain barrier, which was demonstrated again in animal studies, whereby mice injected with ADAMTS13 demonstrated reduced rates of blood brain barrier (BBB) permeability even in combination with vWF and tPA [73]. This is thought to be mediated by several different mechanisms, including reducing the inflammatory response by reduced Intercellular Adhesion Molecule-1 (ICAM-1) and Matrix Metalloproteinase-9 (MMP-9) expression. ADAMTS-13 has also been evaluated as an adjunct to tPA in other mouse stroke models, further demonstrating reduced infarct volumes and reduced intracranial haemorrhage (ICH) rates compared to tPA alone at 2 and 4 h, suggesting a potential to also lengthen the time window for tPA [74, 75]. Treatment with ADAMTS13 is emerging as a very important and viable treatment target in stroke, especially as an adjunctive treatment.

Similarly, targeting the same pathways, N-acetylcysteine (NAC) is another inhibitor of vWF, which has been shown to degrade vWF multimers in thrombus formation in acute ischaemic stroke. It does so by reducing the disulfide bonds in the vWF multimers, resulting in fragmentation of vWF. The coadministration of NAC and a nonpeptidic GpIIb/IIIa inhibitor, led to improved outcomes in terms of recanalization, ischaemic injury and neurological recovery post-ischaemic stroke in a mouse model without an increase in haemorrhagic transformation [76]. Kim et al. studied the use of a dimeric form of N-acetylcysteine NAC called DiNAC N, N’-Diacetyl-L-cystine [77]. In this study DiNAC was noted to have significant reduction in clot weight and surface area compared to tPA when used on white VWF-platelet-rich arterial clots analysed in vitro. However, systematic review assessing 4 clinical trials assessing NAC in AIS have thus far found inconclusive findings in terms of benefit in NIHSS scores and mRS, and only two of which assessed its role as an adjunctive hyperacute stroke therapy [78]. Further, NAC-Safety, phase 2 clinical trial, assessing safety of NAC administration adjunctive to thrombolysis with Alteplase, was terminated early due to the occurrence for fatal ICH in 2 of the 12 patients enrolled (17%), raising safety concerns in patients with prior antiplatelet use [79]. Further larger trials in defined stroke populations can need to be considered to evaluate its role in hyperacute stroke treatment.

Other therapeutics currently under investigation targeting vWF include, BB-031that inhibits vWF binding to platelet receptors. This is currently being studied in phase 2 clinical trial, The Recanalization in Acute Ischemic StrokE [30] trial (NCT06226805) [80].

### Plasmin

Plasmin itself has been explored for stroke treatments, given its direct action on fibrin compared tissue plasminogen activators. Several recombinant forms of plasmin have been studied in animal models and in vitro studies, including DELTA (K2-5) plasmin and miniplasmin, whilst demonstrating some promise in animal and in vitro models have not progressed to clinical trials [81–83]. Microplasmin, recombinant truncated form of human plasmin, was the main form that progressed to a Phase 2 randomised controlled clinical trials. While it was safe and well tolerated, there with no significant differences in reperfusion rates or clinical outcomes compared to placebo [84]. The key issue identified with the use of plasmin as a fibrinolytic agent for stroke is that plasmin is rapidly neutralised in plasma by inhibitors in particular alpha-2 antiplasmin, and so administration intravenously has not been successful. As such, it has since been studied intra-arterially delivered directly to the clot interface to reduce its interaction with plasma inhibitors in a phase 1/2a open label dose escalation trial [85]. While this treatment was well tolerated, there was no clear dose relationship and efficacy in recanalization was limited compared to the endovascular thrombectomy. Nonetheless, the potential remains for its consideration in medium vessel occlusion or residual thrombus.

### Alpha-2-Antiplasmin Inhibition

Alpha-2 antiplasmin is the most abundant inhibitor of plasmin, present in plasma. As such, this poses an important treatment target in stroke management and has been explored in mouse models of stroke. Alpha-2 antiplasmin inactivation in mouse stroke models has demonstrated reduced infarct sizes and lower rates of symptomatic intracerebral haemorrhage (sICH) compared to tPA [86]. A monoclonal antibody against alpha-2 antiplasmin called TS23 is currently under assessment in a Phase 2 clinical trial in patients with acute ischaemic stroke in the extended time window up to 24 h after stroke onset (“SISTER” trial; NCT05948566).

Activated factor XIII (FXIIIa) is postulated to crosslink alpha-2 antiplasmin (alpha2AP) with fibrin to inhibit fibrinolysis and so can be an indirect target for alpha2 antiplasmin inhibition. On analysis of blood samples from stroke patients, increases in FXIII levels increased alpha2-antiplasmin crosslinking, however, this was lower in patients with haemorrhagic transformation [87]. In a mouse model of stroke, administration of Factor XIII inhibitor reduced infarct size, improved fibrinolysis, microthrombi and reduced deficits by means of reducing alpha2-antiplasmin crosslinking and reduced lysis resistance was measured by ex-vivo lysis with Alteplase and Tenecteplase [88]. It is important to note however the limitation in clinical translation for this, given the administration of FXIIIa inhibitor was prior to the induction of stroke in this model which would not be possible clinically.

### TAFIa Inhibition

Another therapeutic target suggested is the compound DS-1040 which is an inhibitor of activated thrombin-activatable fibrinolysis inhibitor (TAFIa). By inhibiting TAFIa, this compound is thought to facilitate endogenous fibrinolysis [89]. DS-1040 has been studied in a phase 1 placebo controlled clinical trial in patients undergoing endovascular thrombectomy within 8 h of onset, overall demonstrating tolerability and safety. Whilst it met its primary safety endpoint, with no symptomatic ICH in either the treatment or placebo arm, there was a trend towards increased risk of asymptomatic ICH in the treatment arm compared to placebo (37.5% vs. 11.1%) [90].

### PAI-1 Inhibition

Other interesting adjuncts tried including targeting PAI-1. Although Tenecteplase has already been created which displays greater PAI-1 resistance compared to Alteplase, a specific molecule that inhibits PAI-1 that has been tested on mouse models is called PAItrap4 [91]. Overall finding that the administration of PAItrap4 along with tPA reduced the dose required for tPA, while also reducing infarct size, and increasing reperfusion in mouse models without increase in the risk of intracranial haemorrhage. While this is an interesting new target, the importance of PAI-1 as a target is unclear as it is labile and it becomes latent and inactive, making it a less attractive therapeutic target [8, 9].

**Table 3:** Summary of current and emerging therapeutics targeting the fibrinolytic pathway for ischaemic stroke

Lytic/Adjunct	Function/Role in stroke	Significance/Findings	Trial evidence
Plasminogen activators			
Currently licensed for use			
Alteplase	Activates plasminogen to plasmin to break down fibrin	Standard of care for acute ischemic stroke; short half-life, requires infusion.	Phase 3 RCTs, AHA/ASA guidelines
Tenecteplase	Modified tPA with extended half-life, PAI-1 resistance, single bolus dosing	Comparable efficacy to Alteplase; emerging as an alternative for stroke treatment.	Phase 3 RCT, AHA/ASA guidelines
Emerging clinical trial evidence			
Reteplase	Recombinant tPA with double bolus dosing, lower fibrin specificity	Slower lysis time; efficacy in stroke needs further study.	Phase 3 RCT
Pro-urokinase	Recombinant human Pro-urokinase given as bolus followed by infusion	Non-inferiority to Alteplase; signal to lower ICH risk and lower cost	Phase 3 open-label non-inferiority RCT
Desmoteplase	Recombinant plasminogen activator derived from bat saliva; high fibrin specificity	Increased odds of vessel recanalization but no difference in functional independence compared to placebo in 3–9-hour treatment window.	Phase 3 double-blind, placebo-controlled RCT
Low Immunogenic Recombinant Staphylokinase	Recombinant low immunogenic version of Staphylokinase; given as single bolus dose of 10 mg.	Non-inferior to alteplase in the 4.5-hour window	Phase 3, open label, non-inferiority RCT
Emerging Therapies			
Phase 3 RCT evidence			
Tirofiban	GP IIb/IIIa receptor blocker	Improved outcomes adjunctive to thrombolysis and/or thrombectomy	Phase 3 double blinded placebo controlled RCT
Phase 1/2 trial evidence			
DS-1040	Inhibits TAFIa to facilitate endogenous fibrinolysis	Phase 1 trial showed tolerability and safety however not powered for efficacy.	Pre-clinical and Phase 1, Single Blind, Placebo-controlled, Randomized trial
Dornase alfa	Breaks down neutrophil extracellular traps (NETs)	Potentiates tPA-mediated clot lysis; Phase 2 clinical trial demonstrating safety (preliminary)	Pre-clinical evidence, Phase 2 trial completed (Preliminary results; final publication awaited)
TS23	Targets Alpha-2-antiplasmin	Monoclonal antibody under trial; shown to reduce infarct size and sICH in preclinical models.	Pre-clinical evidence, Phase 2 clinical trial underway: (NCT05948566)
BB-031	RNA aptamer targeting vWF-platelet binding	Demonstrating efficacy in preclinical and microfluidic models.	Pre-clinical evidence, Phase 2 clinical trial underway (NCT06226805)
N-Acetylcysteine (NAC)	Degrades vWF multimers in thrombus formation	Improves recanalization and outcomes in mouse models; Phase 2 clinical trial terminated early due to 2 fatal intracranial haemorrhages.	Pre-clinical evidence, Phase 2 clinical trial terminated early due to safety concerns
Pre-Clinical Evaluation			
ADAMTS13	Targets vWF involved in clot formation	ADAMTS13 more effective than tPA in preclinical models; protective effect on BBB.	Pre-clinical evidence
Factor XIII Inhibitor	Reduces alpha-2-antiplasmin crosslinking with fibrin	Inhibition reduces infarct size, improves fibrinolysis in preclinical models.	Pre-clinical evidence

## Other Novel Therapeutics

Additional avenues of investigation underway to improve stroke thrombolysis include nanotechnology, antibody mediated thrombolytic treatment as well as sonothrombolysis. The application of nanotechnology to fibrinolytic therapy in AIS offers the prospect of avoiding systemic risks of thrombolysis, with targeted drug delivery using tPA loaded nanoparticles including an intelligent DNA nanodevice that can release active tPA from thrombin-sensitive DNA nanostructures [92]. Further, shear stress activated nanotherapeutics have also been explored, with favourable findings in the preclinical setting [93, 94]. Overall, although nanotherapeutics have demonstrated considerable promise in the preclinical setting, they are yet to demonstrate the same in human clinical trials [95]. Similarly, targeted drug delivery using an antibody mediated approach has been studied pre-clinically demonstrating efficacy in mouse models as a single-chain urokinase plasminogen activator (scuPA) fused with a single-chain antibody (SCE5) targeting activated glycoprotein IIb/IIIa platelet receptor [96]. On the other hand, sonothrombolysis, although apparently safe and feasible, was not reported to improve thrombolysis based on the results of the Phase 3 CLOTBUST-ER trial [97].

## Conclusion

The checkpoints and inhibitors that balance the opposing pathways involved in thrombosis and fibrinolysis offer potential novel treatment targets in acute ischaemic stroke. While the focus of stroke therapeutics has been historically based on plasminogen activation initially driven by Alteplase (and now Tenecteplase), the evolution of novel adjunctive treatments has allowed the possibility of targeting other structural elements within thrombi to improve thrombolysis efficacy. Clearly, the variability and heterogeneity in clot composition between individuals certainly poses challenges to thrombolysis at a population level. Nonetheless, future advances in stroke therapeutics using newer thrombolytics together with a potential myriad of adjunctive treatments and more targeted delivery systems holds much promise to improve thrombolysis for AIS. 

## Data Availability

No datasets were generated or analysed during the current study.
